# Effectiveness of using representative subsets of global climate models in future crop yield projections

**DOI:** 10.1038/s41598-021-99378-7

**Published:** 2021-10-18

**Authors:** Budong Qian, Qi Jing, Alex J. Cannon, Ward Smith, Brian Grant, Mikhail A. Semenov, Yue-Ping Xu, Di Ma

**Affiliations:** 1 Ottawa Research and Development Centre, Agriculture and Agri-Food Canada, Ottawa, Canada; 2 Climate Research Division, Environment and Climate Change Canada, Victoria, Canada; 3 Rothamsted Research, Harpenden, AL5 2JQ Hertfordshire UK; 4 Institute of Hydrology and Water Resources, Zhejiang University, Hangzhou, China; 5 Present Address: School of Civil Engineering and Architecture, Ningbo Tech University, Ningbo, 315100 China

**Keywords:** Climate change, Computational biophysics, Abiotic

## Abstract

Representative subsets of global climate models (GCMs) are often used in climate change impact studies to account for uncertainty in ensemble climate projections. However, the effectiveness of such subsets has seldom been assessed for the estimations of either the mean or the spread of the full ensembles. We assessed two different approaches that were employed to select 5 GCMs from a 20-member ensemble of GCMs from the CMIP5 ensemble for projecting canola and spring wheat yields across Canada under RCP 4.5 and 8.5 emission scenarios in the periods 2040–2069 and 2070–2099, based on crop simulation models. Averages and spreads of the simulated crop yields using the 5-GCM subsets selected by T&P and KKZ approaches were compared with the full 20-GCM ensemble. Our results showed that the 5-GCM subsets selected by the two approaches could produce full-ensemble means with a relative absolute error of 2.9–4.7% for canola and 1.5–2.2% for spring wheat, and covers 61.8–91.1% and 66.1–80.8% of the full-ensemble spread for canola and spring wheat, respectively. Our results also demonstrated that both approaches were very likely to outperform a subset of randomly selected 5 GCMs in terms of a smaller error and a larger range.

## Introduction

A little more than a decade ago climate scenarios used in climate change impact studies were mostly constrained by the availability of climate change simulations performed by global climate models (GCMs). This limitation resulted in large differences in future climate and crop yield projections^1^. The Task Group on Data and Scenario Support for Impact and Climate Assessment (TGICA) of the Intergovernmental Panel on Climate Change (IPCC) included accessibility as an additional criterion for selecting climate scenarios while also recommending a criterion on representativeness so that climate scenarios should be representative of the potential range of future regional climate. Only in this way can a realistic range of possible impacts be estimated^2^. A typical approach to capture the representativeness of climate scenarios is to use 5 GCMs that represent 5 basic classes of climate changes (relatively cool/wet, cool/dry, middle, hot/wet, and hot/dry). The number of GCMs substantially increased from approximately 40 in the Coupled Model Intercomparison Project Phase 5 (CMIP5)^3^ to around 100 in Phase 6 (CMIP6)^4^. A limitation in AgMIP (Agricultural Model Intercomparison and Improvement Project)^5^ and related studies occurred due to the overwhelming number of possible combinations of individual elements within an integrated assessment framework leading to a prohibitive number of simulations. As a consequence, Ruane and McDermid^6^ presented the Representative Temperature and Precipitation (T&P) GCM Subsetting Approach for selecting a practical subset of GCMs for regional integrated assessment of climate impacts. Coincidentally, a 5-GCM CMIP5 subset was used in the first phase of the Inter-Sectoral Impacts Model Intercomparison Project (ISI-MIP)^7^ for all sectoral impacts assessments owing to the need for consistency across regions and sectors. McSweeney and Jones^8^ found that for many regions and seasons, this subset was likely to underestimate both the total uncertainty in future climate impact, and the proportion of total uncertainty that is attributable to the use of different GCMs. However, the effectiveness of using representative subsets was seldom assessed for either the closeness of the subset averages or spread in relation to the full ensembles for projected crop yields.

In addition to the aforementioned T&P GCM subsetting approach, referred to as the T&P approach hereafter, many methods have been proposed for selecting a small number of climate models in climate change impact studies, although they have seldom been used for crop yield projections. For example, methods have been proposed which preselect models based on their performance in simulating historical climate and model independence^9^. Outside of performance-based selection methods, approaches can be categorized into two types – envelope-based, e.g., McSweeney et al.^10^, and clustering. The T&P approach, which can be traced back to Smith and Hulme^11^, is in principle an envelope-based method as it selects simulations from the high and low end of the range of climate change signals in temperature and precipitation. While simple, this method becomes more complicated to implement if multiple climate variables need to be considered. On the other hand, the clustering method, often using the k-means algorithm^12^, can feasibly deal with multivariate data. For example, Houle et al.^13^ applied k-means clustering to monthly mean temperature and precipitation from 86 GCM simulations from the Coupled Model Intercomparison Project phase 3 (CMIP3). They identified five clusters and used the one closest to the centroid of each cluster as a single representative simulation from each group. However, as k-means clustering attempts to maximize explained variance of an ensemble, it selects members that are representative of high-density regions in climate space. In addition, k-means clustering is unlikely to produce an ordered sequence of solutions (i.e., the 6-member clustering may not include scenarios in the 5-member clustering). Essentially, all selection approaches are designed to identify a small number of GCMs to represent climate change signals from a large number of GCMs that can be used in climate change impact studies. This selection reduces the demand for resources while retaining as much information as possible needed for characterizing the range of uncertainty from GCMs.

To overcome these issues, Cannon^14^ proposed to use the Katsavounidis–Kuo–Zhang (KKZ) algorithm^15^ as an automated and objective procedure. Unlike k-means clustering, the KKZ algorithm recursively selects members that best span the spread of an ensemble rather than finding clusters that best characterize high-density regions of multivariate space. It is deterministic and ordered, incrementally adding scenarios to the ones previously selected. Previous studies ^16,17^ found that the KKZ method performed better than k-means clustering in hydrological impact studies with regard to a smaller subset size with a larger percentage of the full-ensemble range.

In this study, we used the T&P approach and the KKZ algorithm to select 5-GCM subsets from an ensemble of 20 CMIP5 GCMs for crop yield projections at 10 locations across Canada under Representative Concentration Pathways (RCPs) 4.5 and 8.5 in the periods 2040–2069 and 2070–2099. Our objectives were (1) to assess the effectiveness of the 5-GCM subsets in terms of closeness of the subset averages and ranges of projected crop yields to the means and spreads of the full 20-GCM ensemble; (2) to verify whether annual or growing season mean temperature and precipitation are more effective for selection; (3) to investigate the effect of the subset size on their effectiveness; (4) to explore the potential of including more climate variables, such as interannual variability and extremes, in the selection using the KKZ algorithm taking its advantages of dealing with multiple variables.

## Study area and climate data

### Study area

**Figure 1:**
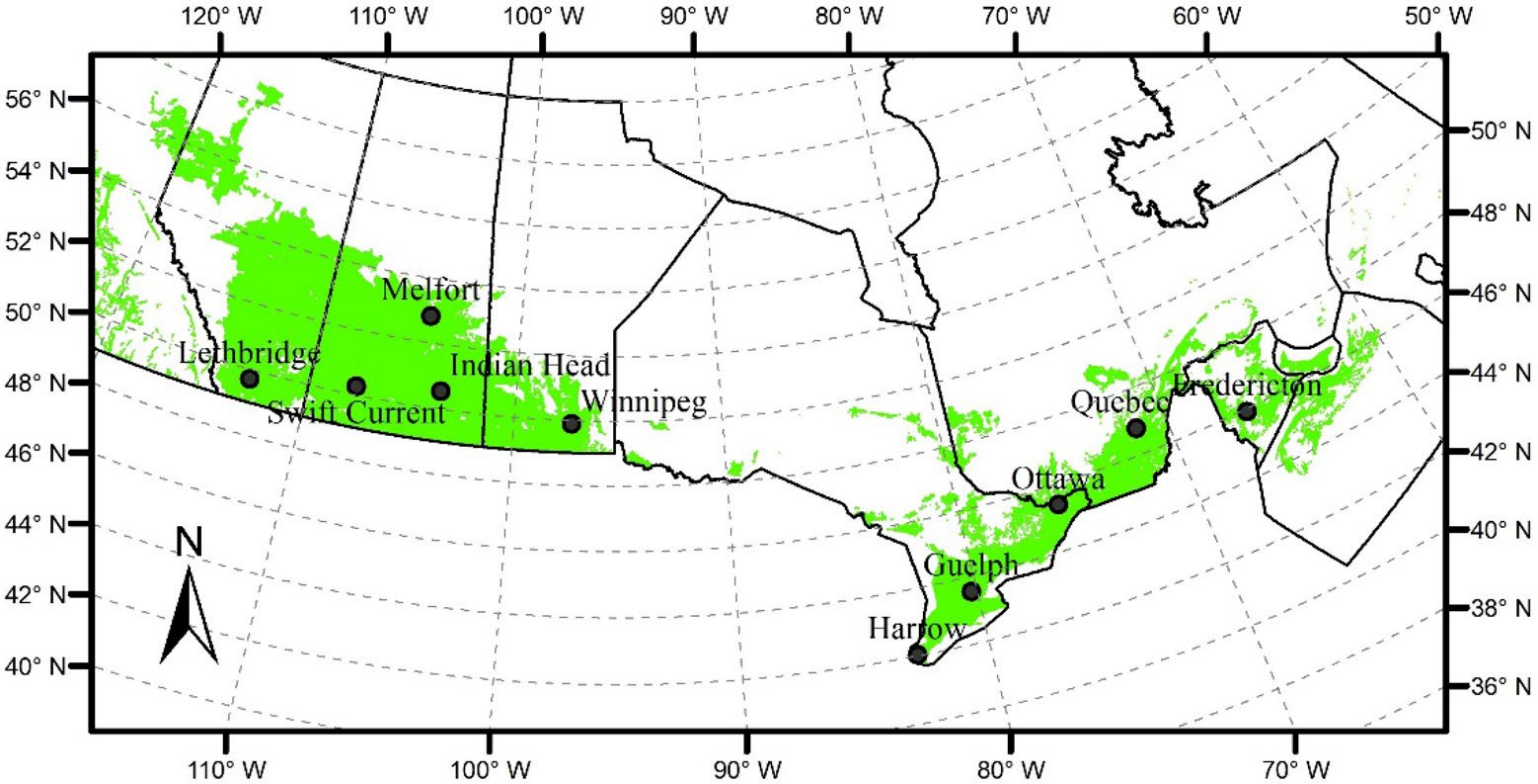
The 10 locations where simulations were performed across the agricultural areas (shaded in green) in Canada.

We selected 10 locations covering diverse climatic regimes and soils in agricultural regions across Canada (Fig. 1) for canola and spring wheat yield simulations. Canola and spring wheat are dominant crops grown in Canada, especially on the Canadian Prairies. Soil data, projected growing season (May 1–August 31) mean temperature and precipitation at these 10 locations for the mid-century (2040–2069) and the late-century (2070–2099) periods under RCP4.5 and RCP8.5 are shown in Supplementary Table S1.

### Climate data

Historical daily maximum temperature (T_max_), daily minimum temperature (T_min_), and daily precipitation (Prec) for 1971–2000 observed at the 10 locations were obtained from Environment and Climate Change Canada’s National Climate Data and Information Archive. Daily global solar radiation (Rad) data were extracted from a high-resolution global dataset of meteorological forcings for land surface modelling^18^ because Rad was either not observed or observations were incomplete at most locations. The observed data were used to bias correct and downscale the GCM simulations using a multivariate form of quantile mapping^19,20^ that first corrects GCM marginal distributions and multivariate dependence structure between sites and variables to match the 1971–2000 historical observations; and, second, preserves GCM-projected changes in quantiles in future periods. Daily outputs of T_max_, T_min_, Prec, and Rad from the 20 GCMs in the CMIP5 archive for two future periods of 2040–2069 and 2070–2099 under the forcing scenarios RCP4.5 and RCP8.5 were used as the full ensemble in this study. The same 20 GCMs were previously used to estimate crop yield projections for Canada^21^ and to quantify uncertainties in crop yield projections^22^. Bias-corrected and downscaled GCM data including daily T_max_, T_min_, Prec, and Rad were used to drive the crop models, as well as for selecting GCMs for the subsets.

## Results and discussion

### Regional averages

Regional averages, i.e., the averages across the 10 locations, of relative absolute error (RAE, %) and relative range (RR, %) of the 5-GCM subsets selected by five methods relative to the 20-GCM CMIP5 ensemble for projected canola and spring wheat yields are shown in Table 1. The averages of RAE are often smaller for spring wheat than canola, although they varied with the selection methods and the scenarios. On the other hand, the averages of RR are slightly larger for canola than spring wheat. When averaged over the four scenarios, using growing season mean temperature and precipitation resulted in a more effective estimation of the ensemble means and spreads in the projected crop yields than using annual values for both the T&P approach and the KKZ algorithm (see Methods). That is, the T&Pg method and the KKZg method resulted in better expectations than their counterparts T&Pa and KKZa. Besides, the T&Pg method led to a smaller RAE than the KKZg method, 2.9% vs. 4.4% for canola and 1.7% vs. 2.0% for spring wheat. In contrast, the KKZg method led to a larger RR than the T&Pg method, e.g., 88.0% vs. 61.8% for canola and 80.8% vs. 68.3% for spring wheat.

**Table 1:** Averages across the 10 locations of relative absolute error (RAE, %) and relative range (RR, %) of the 5-GCM subsets selected using five methods relative to the 20-GCM CMIP5 ensemble for projected canola and spring wheat yields.

Scenario	Method	Canola		Spring Wheat	
		RAE	RR	RAE	RR
RCP4.5 2040–2069	T&Pa	2.5	48.3	2.5	48.7
	T&Pg	2.6	52.2	2.2	66.5
	KKZa	3.1	70.2	1.2	66.9
	KKZg	3.6	86.4	2.5	75.6
	KKZgv	5.2	95.1	2.3	72.6
RCP4.5 2070–2099	T&Pa	4.9	64.2	3.0	48.0
	T&Pg	3.7	54.3	1.6	65.0
	KKZa	4.6	78.2	2.4	58.9
	KKZg	4.4	90.5	1.8	84.8
	KKZgv	2.2	94.2	1.5	79.3
RCP8.5 2040–2069	T&Pa	6.6	72.3	1.4	66.1
	T&Pg	2.0	72.3	1.1	65.7
	KKZa	2.8	86.6	1.7	62.4
	KKZg	3.6	84.4	1.5	77.7
	KKZgv	4.7	88.7	1.5	75.2
RCP8.5 2070–2099	T&Pa	4.9	68.5	1.8	75.3
	T&Pg	3.3	68.5	1.7	75.8
	KKZa	5.6	93.3	1.8	76.3
	KKZg	6.0	90.5	2.0	85.2
	KKZgv	2.3	91.1	0.7	80.9
Average	T&Pa	4.7	63.3	2.2	59.5
	T&Pg	2.9	61.8	1.7	68.3
	KKZa	4.0	82.1	1.8	66.1
	KKZg	4.4	88.0	2.0	80.8
	KKZgv	3.6	91.1	1.5	77.0

The effectiveness of using a representative subset of large multi-GCM ensembles for crop yield projections has seldom been evaluated. However, the relative ranges of the subsets in relation to the full-ensemble spreads for crop yields are in line with the findings in previous studies on hydrological impacts. For example, Chen et al.^23^ found that a 7-simulation subset selected by the KKZ algorithm had a 86.1% coverage of the spread in a 54-simulation ensemble for estimating the times to end of the flood and peak discharge, while it produced a 38.8% coverage for peak discharge and spring mean discharge in a watershed in Canada. In their study, 54 simulations consisting of climate projections by 28 CMIP5 GCMs under RCP4.5 and RCP8.5 were used, rather than GCMs only. Ross and Najjar^16^ found that the percentage of the spread covered by a 5-GCM subset selected by the KKZ algorithm varied from around 20% to over 75% in a 29-GCM ensemble for changes in seasonal runoff in five different watersheds in the United States. Ross and Najjar^16^ further noted that while the KKZ method generally performed well, the results from it and other methods varied somewhat unpredictably based on region and number of models chosen.

While the coverage of the spreads was often assessed in previous studies, closeness of the subset averages to the full-ensemble means was seldom evaluated. Our results indicated that there was a relatively small error, on average, when the subset averages were used to represent the full-ensemble means, especially considering that the mean yield measurement error in wheat field experiments was reported as ± 13.5% ^24^. The T&P approach obtained, on average, a slightly smaller error in relation to the full-ensemble means than the KKZ algorithm while the KKZ algorithm often resulted in a noticeably larger coverage of the full-ensemble spreads than the T&P approach. These differences could be related to the method used in the T&P approach to choose a representative model for each of the five groups, i.e., picking a model that is closest to the center of each group. In addition, the results also showed that selections based on growing season mean temperature and precipitation were, in most cases, more effective than using annual mean temperature and precipitation. This could be due to the fact that growing season temperature and precipitation are more influential than annual values in driving crop yield projections.

The probabilities (*p*-value) of a randomly selected 5-GCM subset to outperform the 5-GCM subsets selected using five methods in terms of a smaller RAE, a larger RR, or both a smaller RAE and a larger RR for canola and spring wheat are shown in Table 2. The probabilities varied with selection methods and the scenarios. T&Pg and KKZg performed better than their annual counterparts, and a randomly selected 5-GCM subset had a slightly smaller probability to outperform T&Pg than KKZg for ensemble means but this was reversed for the ranges. A randomly selected 5-GCM subset had very little chance to outperform KKZg in terms of a smaller RAE and a larger RR. This probability (*p*_*er*_) was only 0.033 for canola and 0.021 for spring wheat. It was 0.125 and 0.081, respectively for canola and spring wheat, for a randomly selected subset to outperform T&Pg. The probabilities estimated by using a total of 155,504 5-GCM subsets were almost identical to the values in Table 2. A comparison of the probabilities estimated by using randomly selected 10,000 5-GCM subsets and the 15,504 5-GCM subsets from all combinations for the selection method KKZgv is shown in Supplementary Table S2 as an example.

**Table 2:** The probability (*p*-value) of a randomly selected 5-GCM subset to outperform the 5-GCM regional subset selected using five methods in terms of a smaller RAE (*p*_*e*_), a larger RR (*p*_*r*_), or both a smaller RAE and a larger RR (*p*_*er*_) for canola and spring wheat.

Scenario	Method	Canola			Spring Wheat		
		*p*_*e*_	*p*_*r*_	*p*_*er*_	*p*_*e*_	*p*_*r*_	*p*_*er*_
RCP4.5 2040–2069	T&Pa	0.187	0.906	0.179	0.698	0.897	0.650
	T&Pg	0.207	0.839	0.187	0.568	0.334	0.207
	KKZa	0.313	0.322	0.114	0.075	0.321	0.029
	KKZg	0.405	0.068	0.041	0.698	0.077	0.056
	KKZgv	0.640	0.007	0.006	0.608	0.138	0.090
RCP4.5 2070–2099	T&Pa	0.475	0.487	0.250	0.850	0.870	0.736
	T&Pg	0.296	0.747	0.230	0.275	0.298	0.064
	KKZa	0.440	0.241	0.118	0.681	0.461	0.294
	KKZg	0.409	0.076	0.042	0.392	0.024	0.009
	KKZgv	0.046	0.029	0.003	0.216	0.111	0.022
RCP8.5 2040–2069	T&Pa	0.378	0.349	0.166	0.175	0.302	0.073
	T&Pg	0.110	0.474	0.064	0.049	0.315	0.021
	KKZa	0.602	0.026	0.020	0.357	0.432	0.190
	KKZg	0.131	0.000	0.000	0.228	0.026	0.008
	KKZgv	0.438	0.007	0.003	0.224	0.058	0.018
RCP8.5 2070–2099	T&Pa	0.143	0.412	0.081	0.140	0.175	0.038
	T&Pg	0.034	0.412	0.019	0.118	0.163	0.030
	KKZa	0.201	0.036	0.003	0.140	0.157	0.033
	KKZg	0.234	0.139	0.050	0.198	0.056	0.012
	KKZgv	0.004	0.095	0.000	0.001	0.101	0.000
Average	T&Pa	0.296	0.539	0.169	0.466	0.561	0.374
	T&Pg	0.162	0.618	0.125	0.253	0.278	0.081
	KKZa	0.389	0.156	0.064	0.313	0.343	0.137
	KKZg	0.295	0.071	0.033	0.379	0.046	0.021
	KKZgv	0.282	0.035	0.003	0.262	0.102	0.033

### Location differences

**Figure 2:**
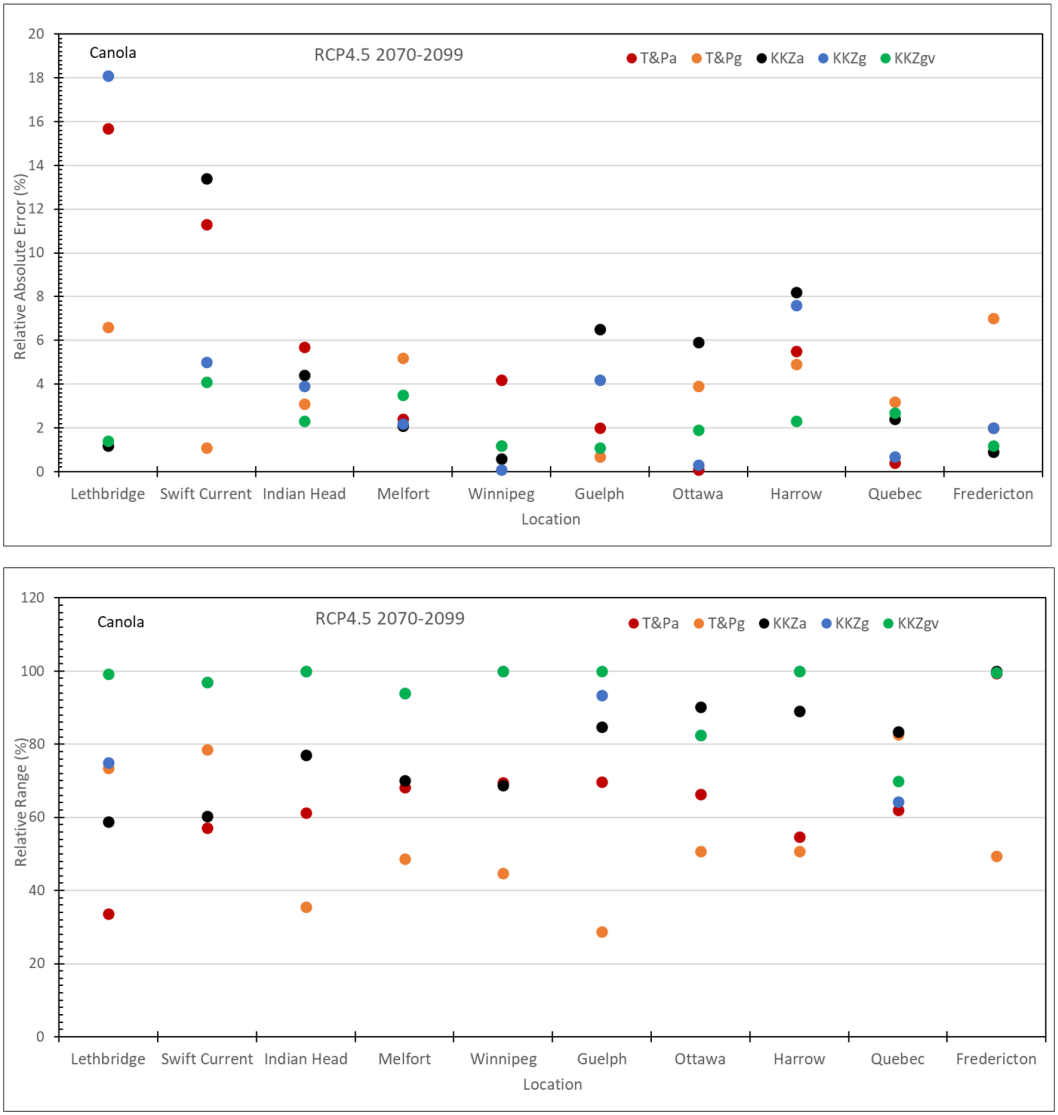
Relative absolute error and relative range of simulated canola yields across the 10 locations for the 5-GCM subset selected using five methods in comparison to the 20-GCM CMIP5 ensemble under RCP4.5 in 2070–2099.

**Figure 3:**
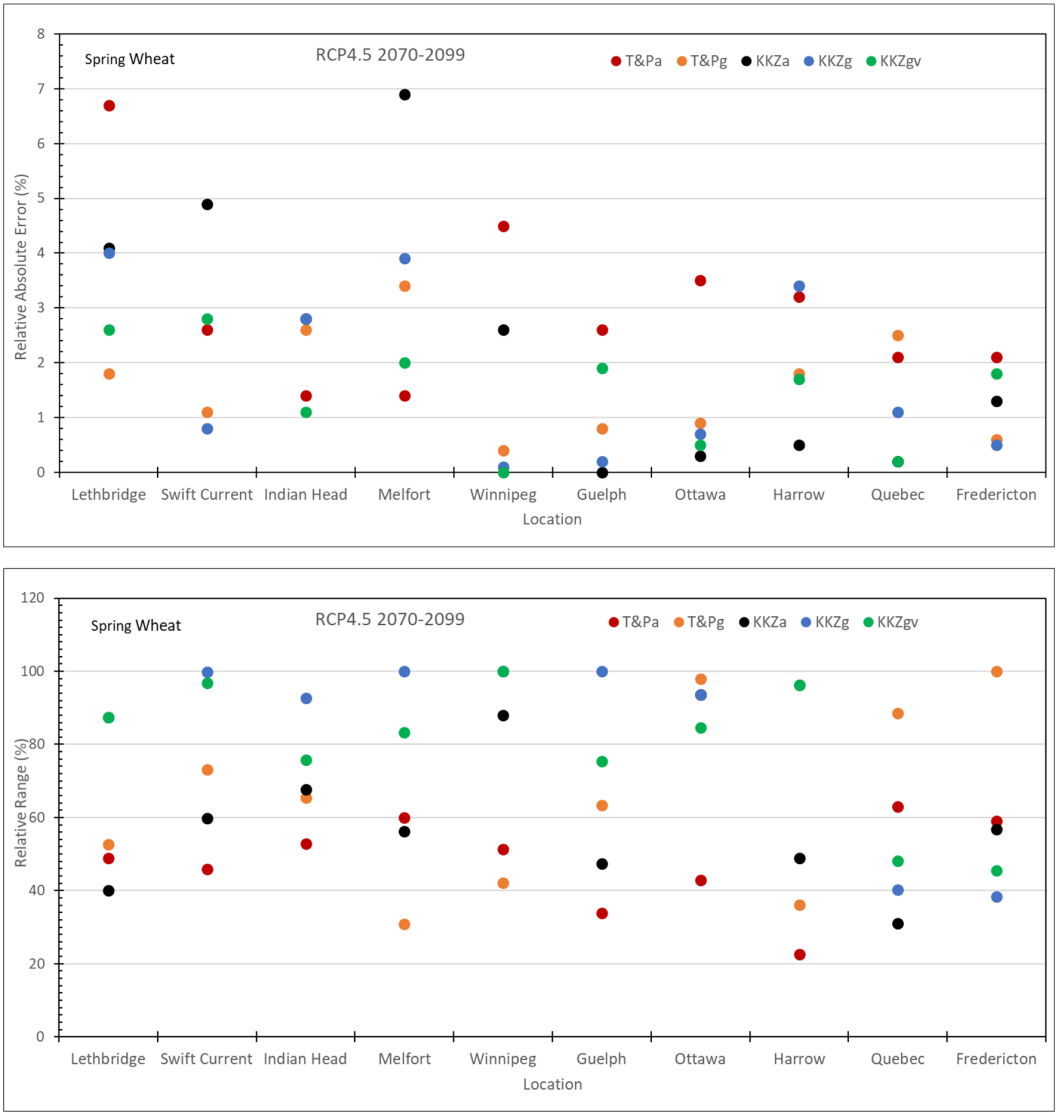
Relative absolute error and relative range of simulated spring wheat yields across the 10 locations for the 5-GCM subset selected using five methods in comparison to the 20-GCM CMIP5 ensemble under RCP4.5 in 2070–2099.

The effectiveness of the subsets to capture the means and spreads of the full ensemble varied significantly across the 10 locations. Although the KKZg method performed better than other three methods, i.e., T&Pa, T&Pg and KKZa, overall, none of the methods consistently outperformed the others for all locations. Relative absolute error and relative range of simulated yields across the 10 locations for the 5-GCM subset selected using different methods in comparison to the 20-GCM CMIP5 ensemble under RCP4.5 in 2070–2099 are shown in Fig. 2 for canola and Fig. 3 for spring wheat, as examples. The relative absolute error for canola in Fig. 2 is mostly smaller than 8% with only two locations larger than 10% while the relative range is mostly over 60% except for the T&P approach, with values smaller than 50% at some locations. The relative absolute errors for spring wheat in Fig. 3 are smaller than 7% and not larger than 4% for both methods based on growing season temperature and precipitation, i.e., T&Pg and KKZg. The relative range is mostly larger than 90% for KKZg but it is only around 40% at two locations, Quebec and Fredericton in eastern Canada where it is over 80% for T&Pg. It is not surprising that larger values of RAE, e.g., for canola at Lethbridge and Swift Current, were related to smaller values of the full-ensemble means. This can also explain the overall smaller RAEs for spring wheat than canola as simulated spring wheat yields were much higher than canola. On the other hand, smaller values of RR could often be linked to the smaller ranges of the full ensemble at the locations such as Quebec and Fredericton in eastern Canada where temperature and precipitation were adequate for canola and spring wheat and thus crop yields were not very sensitive to simulated temperature and precipitation by the GCMs. Mean and range (maximum and minimum) of simulated 30-year averages for canola and spring wheat yields across the 10 locations in Canada using climate scenarios from the 20-GCM CMIP5 ensemble under RCP4.5 in 2070–2099 are shown in Supplementary Figure S1.

### Effect of subset size

**Figure 4:**
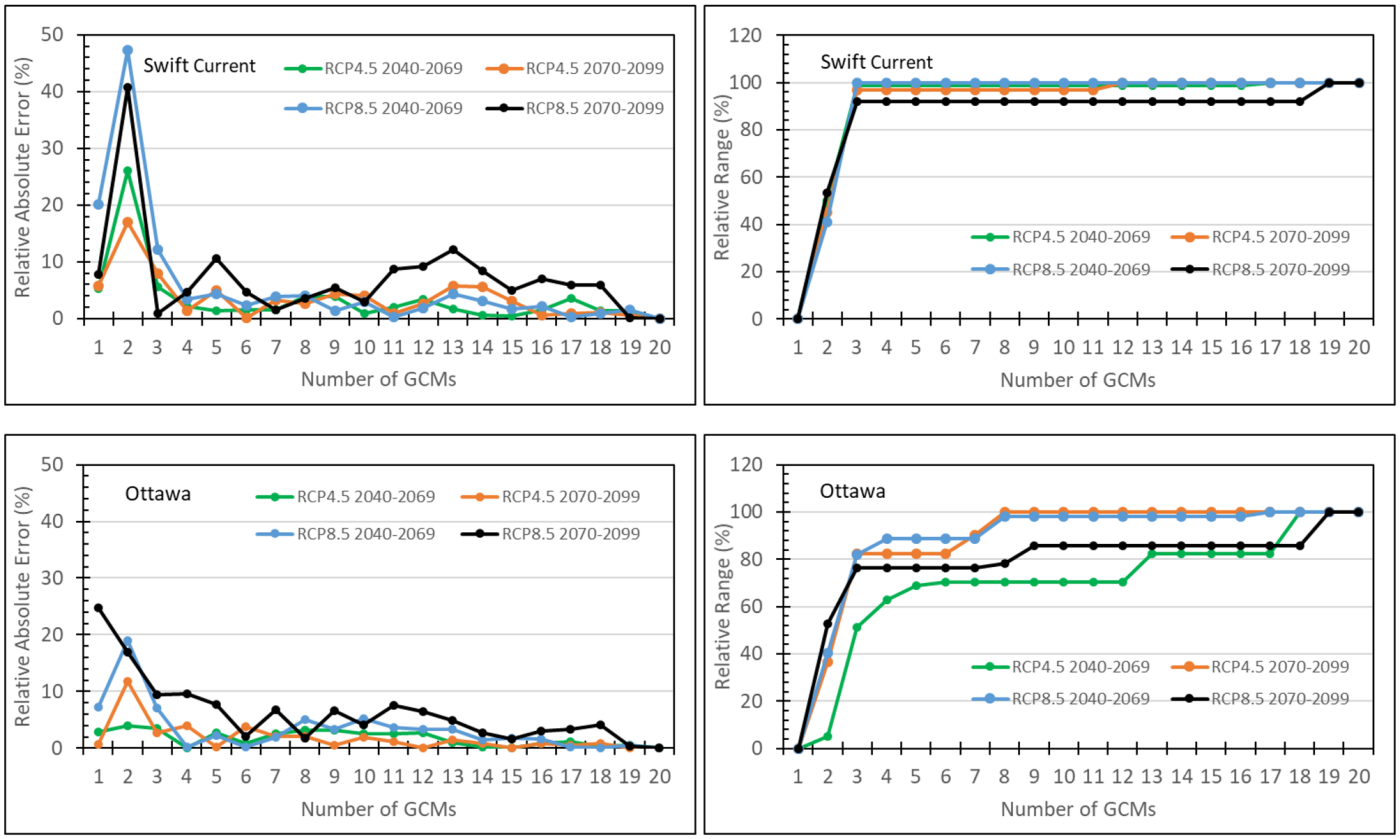
Relative absolute error and relative range of simulated canola yields at Swift Current and Ottawa varying with size of the subset selected using the KKZg method to represent the 20-GCM CMIP5 ensemble under four climate scenarios.

**Figure 5:**
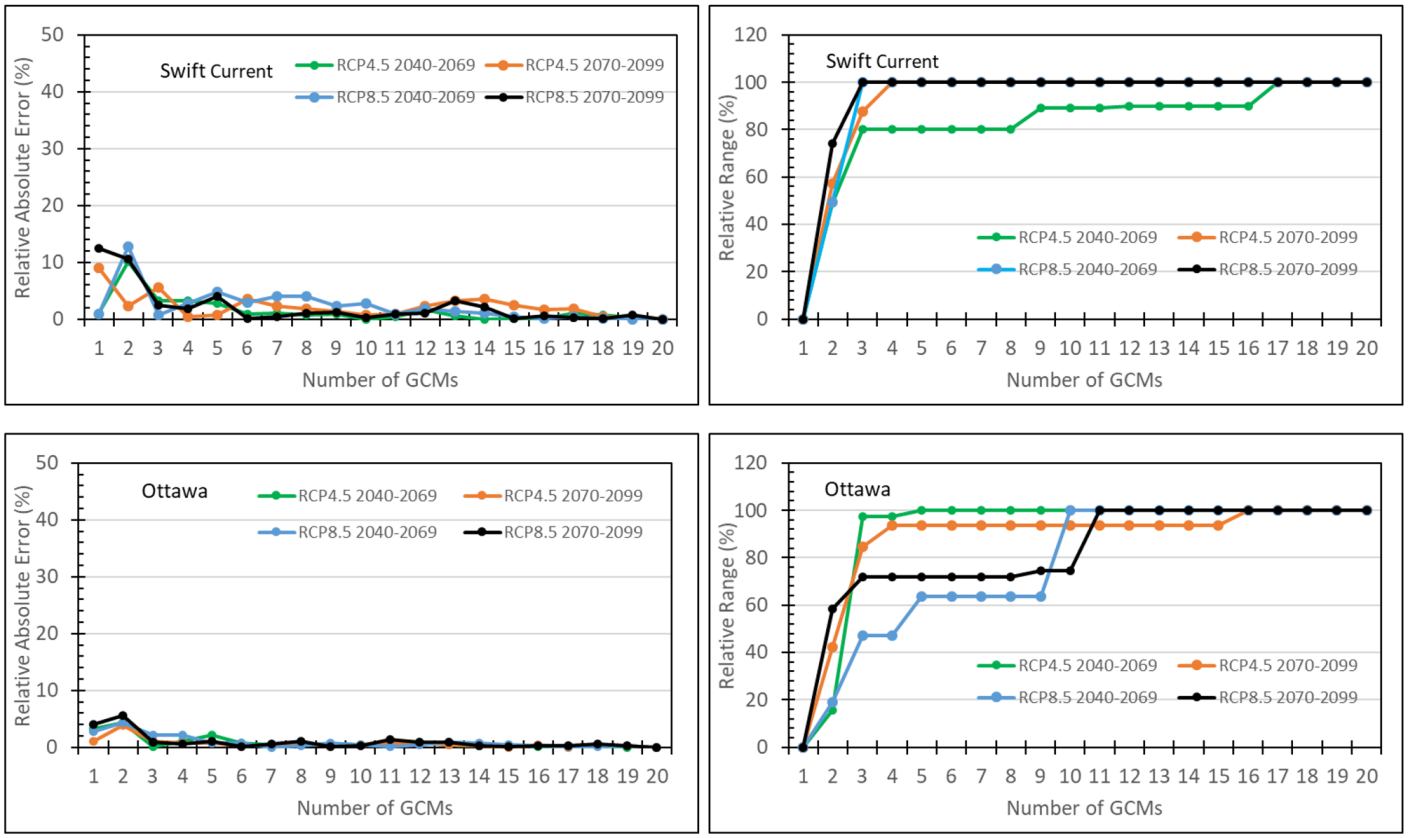
Relative absolute error and relative range of simulated spring wheat yields at Swift Current and Ottawa varying with size of the subset selected using the KKZg method to represent the 20-GCM CMIP5 ensemble under four climate scenarios.

A 5-GCM subset was selected and evaluated in this study because the T&P approach is more practical for selecting a 5-GCM subset and a 5-GCM subset has been often employed in climate change impact studies ^6,7,13^ to represent 5 basic classes of climate change signals. However, it is interesting to investigate the effect of subset size. The KKZ algorithm is not only intended to best span the spread of an ensemble but also to incrementally add members to the subsets previously selected. This implies that the relative range continuously increases with the subset size until it reaches 100%. However, the relative absolute error may not necessarily continuously decrease with the size of the subsets, although RAE is often smaller when the subset size is larger. Figure 4 and Fig. 5 demonstrate how RAE and RR changed with the subset size selected using the KKZg method for canola yield and spring wheat yield under four climate scenarios at Swift Current on the Canadian Prairies and Ottawa in eastern Canada. In these cases, RR often reached a plateau with the subset size of 3 and meanwhile RAE was the largest when the subset size was below 3. Based on these observations, a minimum of 3 GCMs in a subset would be required in order to be representative to the full ensemble in terms of producing the full-ensemble mean and spread.

As the T&P approach primarily deals with mean temperature and precipitation, the subset size would have to be increased to 9 if a third climate parameter, such as the standard deviation of growing season temperature to reflect variability, were introduced in the selection. It might become more complicated if more climate variables need to be considered. The KKZ algorithm, on the other hand, can easily handle multivariate data, thus more climate variables may be introduced in the selection. For example, interannual variability may play an important role in crop yields. In the KKZgv method, we included standard deviations of growing season mean temperature and precipitation in addition to the means used in the KKZg method. Results based on the KKZgv method are included in Tables 1 and 2 and Figs. 2 and 3. The KKZgv method improved, on average, the effectiveness of a 5-GCM subset with a smaller RAE and a larger RR for canola and a smaller RAE but a slightly decreased RR for spring wheat than the KKZg method without taking variability into account in selection. Heat stress can reduce crop yields, and we tried to include the number of days with daily maximum temperature exceeding 29.5 °C during the growing season in the selection in addition to growing season mean temperature, precipitation and their standard deviations (results not shown). However, the inclusion of the number of hot days did not improve the effectiveness of the 5-GCM subsets in yield projections for either canola or spring wheat. Therefore, identifying climate sensitivity indices, as suggested by Semenov and Stratonovich ^25^, could be critical but also challenging for selecting subsets in crop yield projections for different crops and regions around the world, although growing season mean temperature and precipitation appeared relatively effective for spring crops in Canada.

### Implications

Based on the results from this study, using a small subset of GCMs, e.g., 5 GCMs, in crop yield projections can be effective in representing a full GCM ensemble, in terms of a small error to the full-ensemble mean and a large portion of the full-ensemble spread. These results are in line with previous studies for hydrological projections^16,23^. However, the performance of the subsets selected by two approaches varied largely across the 10 locations in Canada spanning diverse climates for two spring crops. The spatial differences may be related to the crop yield responses to local climate, more specifically crop yield limiting factors. For example, water stress is often a dominant limiting factor to crop yields on the Canadian Prairies due to the lack of precipitation but much less water stress occurs to spring crops in eastern Canada with sufficient precipitation. Therefore, climate variables used for subset selections might need to reflect regional limiting factors to crop yields. We found in this study that using growing season mean temperature and precipitation often resulted in a better performance than using the annual mean. More studies with distinct climate regimes and cropping systems in other regions around the world may provide a better understanding of the effectiveness of using subsets of GCMs in crop yield projection.

Two well-evaluated crop models, CERES and CROPGRO, in the widely used crop modelling package DSSAT were used in this study. Large uncertainty in crop modelling is well recognized ^26^. Therefore, future studies employing multiple crop models may be helpful for further assessing the effectiveness of using subsets to represent GCM ensembles in climate change impact studies. In addition to long-term means, changes in interannual yield variability may also need to be assessed. In this study, we used the closeness to the ensemble mean and spread to measure the effectiveness. It might be interesting to evaluate other statistics of the full ensemble, such as 5% and 95% percentiles instead of the full range depending on what information that stakeholders need for making their decision on climate change adaptations.

Nevertheless, using representative subsets of a full GCM ensemble in climate change impact studies is a compromise between resources and information loss due to the overwhelming number of simulations required^6^. Compared to the T&P, multivariate approaches such as the KKZ algorithm, which recursively select members that best span the spread of a full ensemble, might be more feasible for selecting GCM subsets in climate change impact studies. Although pre-determining the size of subsets to meet targeting accuracy and spread is challenging, it is recommended to use subsets as large as possible whenever resources permit, especially if the range of the outcome is critical, given that the relative range continuously increases with the subset size.

## Conclusions

Based on simulated canola and spring wheat yields in two future periods, 2040–2069 and 2070–2099 under RCP4.5 and RCP8.5, using 5-GCM subsets selected by the T&P approach and the KKZ algorithm, the subset averages and ranges could well represent the means and spreads of the full 20-GCM CMIP5 ensemble.

Subset selections based on growing season mean temperature and precipitation were overall more effective than those based on annual mean temperature and precipitation. While the T&P approach could often lead to a smaller relative absolute error in relation to the full-ensemble means than the KKZ algorithm, the latter could cover a larger portion of the full-ensemble spread than the former. The KKZ algorithm had a higher probability than the T&P approach to outperform a randomly selected 5-GCM subset in terms of both a smaller error to the full-ensemble mean and a larger portion of the full-ensemble spread. A minimum of 3 GCMs in a subset would be required to reasonably represent the full ensemble.

More climate variables could be effortlessly incorporated into the selection of subsets with the KKZ algorithm. The effectiveness of the 5-GCM subsets was improved in terms of a smaller relative absolute error to the full-ensemble mean and a larger relative range of the full-ensemble spread for canola and a smaller relative absolute error for spring wheat when interannual variability of growing season mean temperature and precipitation total was incorporated into the selection. However, introducing number of hot days in the selection did not seem to improve the effectiveness in this case. Identifying climate sensitive indices for subset selections could be critical and challenging. Moreover, it is unknown if the effectiveness of the subsets for future crop yield projections would be different when other crop models were used. A study based on multiple crop models may further advance our understanding on the effectiveness of using subsets of a multi-GCM ensemble for future crop yield projections.

## Methods

### Crop simulation

Two crop growth models, the CSM-CROPGRO-Canola model and the CSM-CERES-Wheat model, in the Decision Support System for Agrotechnology Transfer (DSSAT) v4.7^27^, were driven by the bias-corrected and downscaled climate scenarios of the 20 GCMs to simulate crop yields for canola and spring wheat at the 10 locations. Crop models in DSSAT have been widely used in climate change impact studies worldwide, and these two models have been calibrated and evaluated with field experimental data in Canada^28,29^. These models have also been used to project future crop yields in Canada and quantify uncertainties related to climate projections^21,22^. Soil data, crop cultivar parameters, and crop management data are also required as input to these crop models in addition to climate data. Soil data for a representative soil at each location (Supplementary Table S1) were obtained from the Canadian Soil Information System (CanSIS), Soil Landscapes of Canada (SLC), version 3.2^30^. Crop cultivar parameters for spring wheat cultivar AC Barrie and canola cultivar InVigor 5440 calibrated with Canadian data by Jing et al.^28,29^ were used to simulate crop yields as continuous spring wheat and canola. The planting date in simulations for both spring wheat and canola was May 8, approximately one week earlier than for the current-day climate, on average, taking into account the potential for an advance in growing season under the warmer future climate. The potential for the advance in growing season could be more remarkable in the late-Century under RCP8.5 but we used the same planting date for simplicity. Crops were harvested automatically at physiological maturity in all simulations. We simulated only the water-limited yield of the crops grown without nitrogen stress to emphasize the climate impacts. Soil texture may have significant impacts on crop growth and yield in the simulations, therefore simulated crop yields at the selected locations could differ if other soil types were used. All simulations included the direct effects of elevated atmospheric CO_2_ concentration on photosynthesis and transpiration^31^. In the CSM-CROPGRO-Canola model, photosynthesis of sunlit and shaded leaves is computed hourly using the asymptotic exponential response equation, where quantum efficiency and light-saturated photosynthesis rate depend on CO_2_ and temperature^32^. Doubling the ambient CO_2_ concentration may increase the photosynthetic rate by about 30%. In the CSM-CERES-Wheat model, the radiation use efficiency (RUE) is simply modified by a linear function where RUE increases with increasing CO_2_ concentration. Doubling the ambient CO_2_ concentration may increase RUE by about 25%. In these models, crop transpiration is reduced to account for the effects of increased atmospheric CO_2_ concentration on stomatal resistance.

### Selection of GCM subsets

Preselection may be performed to eliminate some models based on their skill in simulating historical climate as well as model independence^9^ although the best performing models in the past do not necessarily produce the most credible projections of future climate. In fact, a “one-model-one vote” model democracy interpretation of GCMs to avoid eliminating individual models for CMIP5 was adopted by IPCC in its Fifth Assessment Report^33^. In this study, we treated climate projections of the 20 CMIP5 GCMs previously used in Qian et al.^21^ equally plausible.

Two approaches, the T&P approach and the KKZ algorithm, were used to select a subset of 5 GCMs from the full ensemble, i.e., 20 CMIP5 GCMs, based on the bias-corrected and downscaled temperature and precipitation at the 10 locations. The subsets were separately selected for the two future periods and two RCPs; thus 4 regional subsets were chosen, using each approach, when the selection was based on regional climate, i.e., averages of temperature and precipitation across the 10 locations were used in the T&P approach while climate variables at the 10 locations were used altogether in the KKZ algorithm.

### The T&P approach

Ruane and McDermid^6^ presented a T&P approach that is built upon the use of climate sensitivity indices^25^ to select a 5-GCM subset based on annual/growing season mean temperature and precipitation changes. As the bias-corrected and downscaled GCM data were used in this study, the baseline climate at a location was not different across the GCMs. Therefore, we selected the subsets based on the 30-year means of both annual and growing season mean temperature and precipitation for the future periods, rather than their changes from the baseline, and considered them as two different methods, T&Pa and T&Pg, respectively. The growing season period from May 1st to August 31st applies to both canola and spring wheat in Canada. Annual and growing season mean temperature and precipitation for a future period under a forcing RCP scenario, averaged across the 10 locations, were calculated for each of the 20 GCMs and further the medians over the 20 GCMs were determined. A given GCM was classified as “relatively cool” when its projected temperature was lower than the median of all GCMs. Similarly, a given GCM was considered as “relatively wet” when its projected precipitation was larger than the full GCM ensemble median. Eventually, each GCM was classified into four basic quadrants: “cool/wet”, “cool/dry”, “hot/wet”, “hot/dry”. A fifth, “Middle”, classification was also introduced to include the models that represent the nexus of these quadrants around the median of the full ensemble. The ensemble standard deviation (σ) of temperature and precipitation was used to classify the GCMs into the “Middle” category if their temperature and precipitation were within ± (ρ × σ) of the median, where ρ is a standard deviation factor designed as a simple measure of spread in order to capture approximately 1/5th (20%) of all GCM projections. This approach targets each quadrant to contain approximately the same number of models to minimize the number of GCMs that each quadrant’s selected model has to represent. We used ρ = 0.5 in the selection with regional climate as recommended by Ruane and McDermid^6^ as projections were often skewed. Once all GCMs were classified into one of the five groups, i.e., “cool/wet”, “cool/dry”, “hot/wet”, “hot/dry”, and “Middle”, one GCM that was in the center of each group was chosen as a representative GCM for the group as described in Ruane and McDermid^6^.

### The KKZ algorithm

The KKZ algorithm^15^ was originally designed for initializing the centroids in k-means clustering. Cannon^14^ introduced the KKZ algorithm for recursively selecting members that best span the spread of an ensemble. To be comparable with the T&P approach, we also used annual and growing season mean temperature and precipitation for selecting the subsets and denote them as two different methods, KKZa and KKZg, respectively. Taking into account the algorithm’s advantage of dealing with multiple variables, we also included a method (KKZgv) that considers interannual variability in terms of the standard deviations in a 30-year period of growing season mean temperature and precipitation. This KKZ algorithm consists of the following four steps:

1. Select the member that lies closest to the ensemble centroid [Eq. (1)] as the first subset member;
  1 $$ \left( X \right)_{.p} = \frac{1}{N}\mathop \sum \limits_{i = 1}^{N} \left( X \right)_{ip} $$
  where $\left( X \right)_{.p}$ is the ensemble centroid; *N* is the number of cases in the full ensemble, i.e., 20 in this study; *p* = 1, *P;* and *P* is the number of variables; $\left( X \right)_{ip}$ is the value of the *p*th variable for the *i*th member; *P* varies from 20 (annual/growing season mean temperature and precipitation, two variables at each of the 10 locations) in KKZa and KKZg to 40 (annual/growing season mean temperature and precipitation and their standard deviations, four variables at each of the 10 locations) in KKZgv. All variables were standardized to zero mean and unit standard deviation. The distance (D_*i*_) of the *i*th member to the ensemble centroid is calculated using Eq. (2).
  2 $$ D_{i} = \mathop \sum \limits_{p = 1}^{P} \left[ {\left( X \right)_{ip} - \left( X \right)_{.p} } \right]^{2} $$
2. Select the member that lies farthest from the first subset member as the second subset member. The distance (D_ik_) of the *i*th ensemble member to the *k*th subset member (*k*′ ∈ [1,N], i.e., the *k*th subset member is the *k*′th member of the full ensemble) is calculated using Eq. (3).
  3 $$ D_{ik} = \mathop \sum \limits_{p = 1}^{P} \left[ {\left( X \right)_{ip} - \left( X \right)_{k^{\prime}p} } \right]^{2} $$
3. To select the next subset member,
  (i) calculate distances from each remaining ensemble member to the previously selected subset members using Eq. (3);
  (ii) associate each remaining ensemble member with the minimum distance calculated in step 3(i); and
  (iii) select the ensemble member with the maximum distance from step 3(ii) as the next subset member.
4. Repeat step 3 until 5 subset members are selected. The KKZ algorithm can continue to be run for numbers of subset members greater than 5. In this case, it provides a deterministic and ordered subset of the specified size.

### Quantify the effectiveness of the selected subsets

As the subsets were selected to represent the full ensemble, we quantify their effectiveness in terms of a small relative absolute error (RAE) [Eq. (4)] for the subset average relative to the full-ensemble mean and a large relative range (RR) [Eq. (5)] for the subset in comparison to the full-ensemble spread of simulated crop yields. Spread and range are exchangeable here to quantify the uncertainty related to GCMs in simulating crop yields as the difference between the maximum and the minimum yield values in the full ensemble or the subsets. All ensemble means and ranges are all based on the 30-year means of simulated crop yields for a future scenario. Let *Y*_*enf*_ and *Y*_*ens*_ be the average yield of the full ensemble and the subset, respectively,

4 $$ RAE = \frac{{\left| {Y_{ens} - Y_{enf} } \right|}}{{Y_{enf} }} \times 100\% $$

And let *Y*_*maxf*_, *Y*_*minf*_, and *Y*_*maxs*_, *Y*_*mins*_ be the maximum and minimum average yields across the members of the full ensemble and the subset, respectively,

5 $$ RR = \frac{{Y_{maxs} - Y_{mins} }}{{Y_{maxf} - Y_{minf} }} \times 100\% $$

A resampling test was performed to estimate the probabilities that a randomly chosen 5-GCM subset would outperform the 5-GCM subsets selected using the T&P approach and the KKZ algorithm by having a smaller RAE, a larger RR and both a smaller RAE and a larger RR. The randomly chosen subsets were drawn from the 20-GCM full ensemble without replacement 10,000 times; thus each subset could consist of five different GCMs. Let *Y*_*ens′*_, *Y*_*maxs*′_, and *Y*_*mins*′_ be the subset average, and maximum and minimum yields across the 5 members of the randomly chosen subset, respectively,

6 $$ N_{e} = \mathop \sum \limits_{i = 1}^{10000} (1, if \left| {Y_{ens^{\prime}} - Y_{enf} } \right| < \left| {Y_{ens} - Y_{enf} } \right|; \, 0,{\text{ otherwise}}) $$

7 $$ N_{r} = \mathop \sum \limits_{i = 1}^{10000} (1, if \left| {Y_{maxs^{\prime}} - Y_{mins^{\prime}} } \right| > \left| {Y_{maxs} - Y_{mins} } \right|;{ }0,{\text{ otherwise}}) $$

8 $$ N_{er} = \mathop \sum \limits_{i = 1}^{10000} (1, if \left| {Y_{{ens^{\prime}}} - Y_{enf} } \right|\left\langle {\left| {Y_{ens} - Y_{enf} } \right| and \left| {Y_{maxs^{\prime}} - Y_{mins^{\prime}} } \right|} \right\rangle \left| {Y_{maxs} - Y_{mins} } \right|;{ }0,{\text{ otherwise}}) $$

9 $$ p_{e} = \frac{{N_{e} }}{10000} $$

10 $$ p_{r} = \frac{{N_{r} }}{10000} $$

11 $$ p_{er} = \frac{{N_{er} }}{10000} $$

where *p*_*e*_, *p*_*r*_, and *p*_*er*_ are the probabilities that a randomly selected 5-GCM subset would likely outperform the 5-GCM subset selected using the T&P approach or the KKZ algorithm by a smaller *RAE*, a larger *RR* and both a smaller *RAE* and a larger *RR*. These probabilities can also be estimated by using a total of 15,504 5-GCM subsets, i.e., all 5-GCM combinations from a 20-GCM ensemble, in the place of randomly selected 10,000 5-GCM subsets.

## Supplementary Information

Supplementary Information.
