# Supplementary material for: Effectiveness of using representative subsets of global climate models in future crop yield projections
Source: Sci Rep. 2021 Oct 18;11:20565. doi: 10.1038/s41598-021-99378-7 (PMC8523532; doi:10.1038/s41598-021-99378-7)
Supplement: Supplementary file 1 — Supplementary Information. [file 41598_2021_99378_MOESM1_ESM.docx]

Effectiveness of using representative subsets of global climate models in future crop yield projections

Budong Qian^1*^, Qi Jing^1^, Alex J. Cannon^2^, Ward Smith^1^, Brian Grant^1^, Mikhail A. Semenov^3^, Yue-Ping Xu^4^, Di Ma ^1,4^

**Supplementary**

Table S1. Soil characteristics and projected growing season (May 1^st^ – August 31^st^) mean temperature and precipitation at 10 locations across Canada

| Location | Soil^1^ | | | Climate^2^ | | | | | | | |
| --- | --- | --- | --- | --- | --- | --- | --- | --- | --- | --- | --- |
|  |  |  |  | RCP4.5 | | | | RCP8.5 | | | |
|  |  |  |  | 2040-2069 | | 2070-2099 | | 2040-2069 | | 2070-2099 | |
|  | Series name | Texture | AWC | T (°C) | P (mm) | T (°C) | P (mm) | T (°C) | P (mm) | T (°C) | P (mm) |
| Lethbridge | Lethbridge | Loam | 1.386 | 18.1 | 218.0 | 18.9 | 218.2 | 19.0 | 214.1 | 21.3 | 221.2 |
| Swift Current | Wymark | Loam | 1.416 | 18.1 | 212.7 | 18.8 | 209.2 | 19.0 | 212.5 | 21.3 | 214.2 |
| Indian Head | Indian head | Clay | 1.817 | 18.2 | 262.4 | 19.0 | 260.6 | 19.2 | 261.9 | 21.4 | 264.7 |
| Melfort | Melfort | Silty clay | 1.820 | 17.3 | 248.4 | 18.0 | 250.9 | 18.2 | 252.8 | 20.4 | 251.5 |
| Winnipeg | Red River | Clay | 2.376 | 19.1 | 293.8 | 19.8 | 295.1 | 20.1 | 292.9 | 22.3 | 290.5 |
| Guelph | Burford | Sandy loam | 1.218 | 19.4 | 357.6 | 20.1 | 362.4 | 20.3 | 357.0 | 22.5 | 362.8 |
| Ottawa | Ste. Rosalie | Silty loam | 0.905 | 20.5 | 364.0 | 21.1 | 364.1 | 21.5 | 357.8 | 23.7 | 365.3 |
| Harrow | Tuscola | Silty loam | 1.764 | 22.0 | 359.3 | 22.7 | 356.6 | 22.9 | 354.1 | 25.1 | 352.4 |
| Quebec | Joly | Clay loam | 1.237 | 18.6 | 473.5 | 19.2 | 480.4 | 19.5 | 480.1 | 21.7 | 490.3 |
| Fredericton | Oromocto | Sand | 0.711 | 18.7 | 393.8 | 19.2 | 396.7 | 19.5 | 397.1 | 21.7 | 410.1 |

^1^Soil texture and available water-holding capacity (AWC, mm/cm) are based on the soil profile of 0-100 cm depth.

^2^Projected growing season mean temperature and precipitation total are ensemble means of the 20 CMIP5 GCMs.

Table S2. The probability (*p*-value) of a randomly selected 5-GCM subset (R) to outperform the 5-GCM regional subset selected using the KKZgv method in terms of a smaller RAE (*p_e_*), a larger RR (*p_r_*), or both a smaller RAE and a larger RR (*p_er_*) for canola and spring wheat in comparison with the probability based on all 15504 5-GCM subsets (C) from the 20-GCM ensemble

| Scenario | Method | Canola | | |  | Spring Wheat | | |
| --- | --- | --- | --- | --- | --- | --- | --- | --- |
|  |  | *p_e_* | *p_r_* | *p_er_* |  | *p_e_* | *p_r_* | *p_er_* |
| RCP4.5 2040-2069 | R | 0.640 | 0.007 | 0.006 |  | 0.608 | 0.138 | 0.090 |
|  | C | 0.638 | 0.009 | 0.007 |  | 0.615 | 0.139 | 0.091 |
| RCP4.5 2070-2099 | R | 0.046 | 0.029 | 0.003 |  | 0.216 | 0.111 | 0.022 |
|  | C | 0.049 | 0.031 | 0.004 |  | 0.220 | 0.103 | 0.021 |
| RCP8.5 2040-2069 | R | 0.438 | 0.007 | 0.003 |  | 0.224 | 0.058 | 0.018 |
|  | C | 0.440 | 0.008 | 0.005 |  | 0.228 | 0.061 | 0.019 |
| RCP8.5 2070-2099 | R | 0.004 | 0.095 | 0.000 |  | 0.001 | 0.101 | 0.000 |
|  | C | 0.005 | 0.094 | 0.001 |  | 0.001 | 0.100 | 0.000 |
| Average | R | 0.282 | 0.035 | 0.003 |  | 0.262 | 0.102 | 0.033 |
|  | C | 0.283 | 0.035 | 0.004 |  | 0.266 | 0.100 | 0.033 |


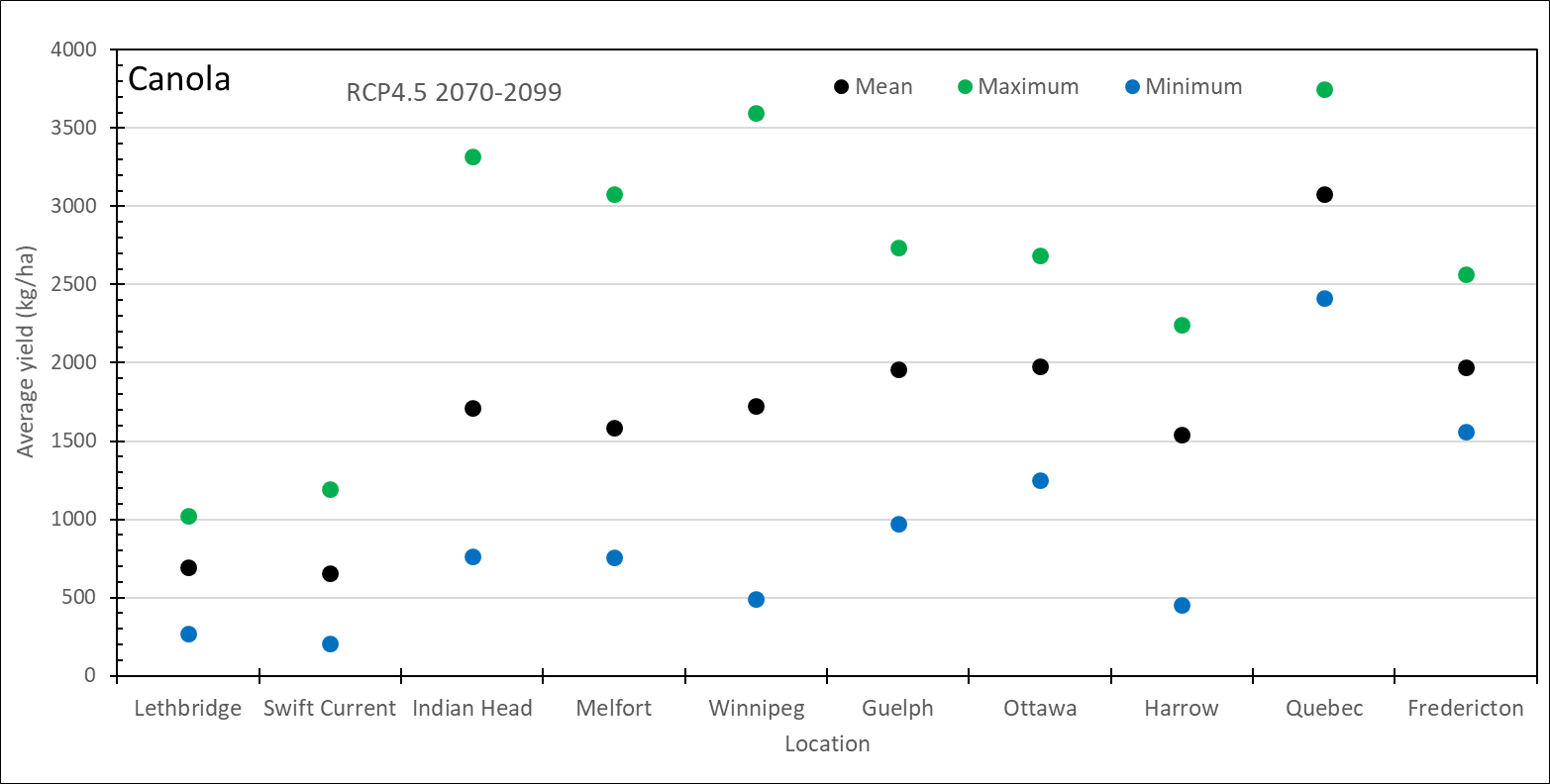


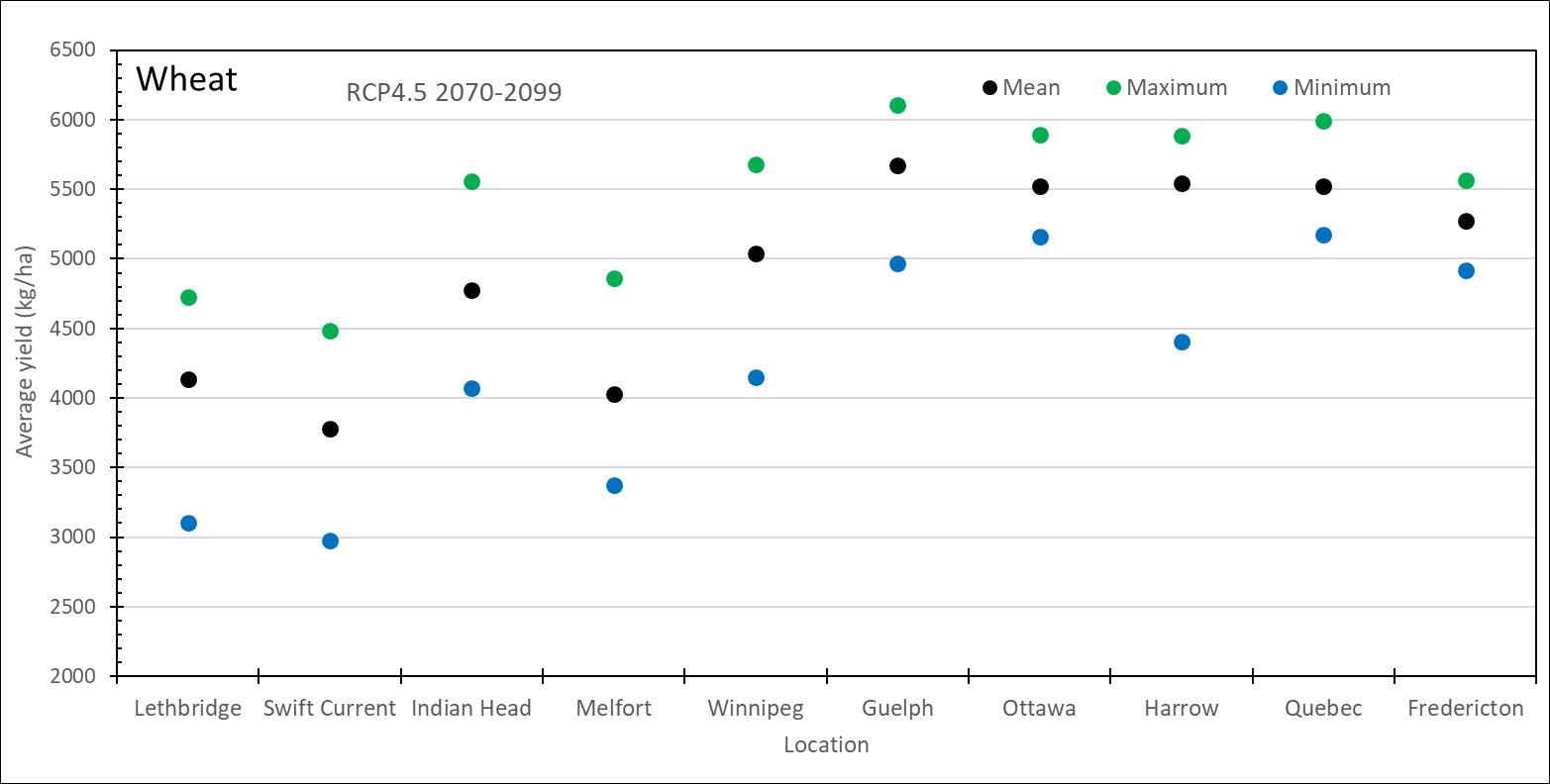


Figure S1. Mean and range (maximum and minimum) of simulated 30-year averages for canola and spring wheat yields across the 10 locations in Canada using climate scenarios from the 20-GCM CMIP5 ensemble under RCP4.5 in 2070-2099
